# Traditional Chinese Herbal Products for Coronary Heart Disease: An Overview of Cochrane Reviews

**DOI:** 10.1155/2012/417387

**Published:** 2012-03-28

**Authors:** Yu Qiu, Hao Xu, Dazhuo Shi

**Affiliations:** ^1^Graduate School, Beijing University of Chinese Medicine, Beijing 100029, China; ^2^Center for Cardiovascular Diseases, Xiyuan Hospital, China Academy of Chinese Medical Sciences, Beijing 100091, China

## Abstract

*Objective*. The aim of this overview was to evaluate and summarize Cochrane reviews of traditional Chinese herbal products (TCHPs) as the treatment for coronary heart disease (CHD). *Methods*. We searched the Cochrane Database that was concerned with the effectiveness of TCHPs for CHD. We also searched the Cochrane Central Register of Controlled Trials. Reviews and primary studies of TCHP as the treatment of any type of CHD were included. Data were extracted according to predefined inclusion criteria by two independent reviewers. *Results*. Six Cochrane reviews were included. They related to a wide range of TCHPs for different types of CHD. Four reviews were concerned with angina pectoris (unstable or stable), one review was concerned with heart failure, and for acute myocardial infarction. No reviews concluded that TCHPs were definitely effective for CHD because of the weak evidence. Eight primary studies were TCHPs from CHD. These studies also maybe result in bias, but better than before. *Conclusion*. Several Cochrane reviews of TCHPs for the treatment of different types of CHD have recently been published. None of these reviews got definite conclusion favoring the effectiveness of TCHPs due to the weak evidence. With the improved quality of the new registered RCTs. The potential role of TCHPs in treating CHD is anticipated to be detected.

## 1. Introduction

Coronary heart disease (CHD) is one of the most dangerous threats to human health, manifested by different clinical types such as angina pectoris, myocardial infarction, heart failure, cardiac arrhythmia, and so forth. Although treated with intensive medication or revascularization therapy, uncontrolled angina and recurrent acute cardiovascular events are still the major problems confronting modern medicine. Traditional Chinese medicine (TCM) has a history of thousands of years and has made great contributions to the health and well-being of the people and to the maintenance and growth of the population [1]. Currently, more than 90% of the urban and rural Chinese population has sought for TCM in their lifetimes [2]. TCM has been studied extensively and seems to be safe and effective in treating CHD [3, 4]. Recently, the potential benefit of integrative Western and Chinese medicine regimen has also been indicated in a large-scale registry study in China [5]. Cochrane reviews are regarded as the highest standard of evidence [6]. They adopt transparent and comprehensive methods of finding all of the relevant evidence. Their quality and reliability are generally higher than any other systematic review because they employ a predefined, rigorous, and explicit methodology. Cochrane reviews are also reviewed and published in advance. Therefore, conclusion made from the overview of Cochrane reviews is more credible. Some Cochrane systematic reviews of traditional Chinese herbal products (TCHPs) for CHD have been conducted in recent years. These reviews provide preliminary evidence of TCHPs benefits to certain CHD patient populations, which call for a comprehensive evaluation on the effectiveness of TCHPs in CHD patients. This overview aims to evaluate and summarize all Cochrane reviews of TCHP as a treatment of CHD critically.

## 2. Methods

We searched the titles and abstracts of all reviews in September 2011 of the Cochrane Database of Systematic Review. The search terms were “Herb* and medic* and heart” and “Herb* and medic* and cardiac” and “Herb* and medic* and circulation” and “Chinese and heart” and “Chinese and cardiac” and “Chinese and circulation.” We read the title and abstract of each retrieved review in order to confirm that the review was relevant. Articles were included if they related to any type of TCHP as a treatment of CHD. Data were extracted according to predefined inclusion criteria by two independent reviewers (Qiu Y. and Xu H.). Disagreements were resolved by discussion between the authors.

We also searched the Cochrane Central Register of Controlled Trials (CENTRAL) in The Cochrane Library Issue 4 of 4, Oct 2011. Studies of TCHP as the treatment of any type of CHD were included. Studies without results were excluded. The methodological quality was assessed using the Cochrane Collaboration risk of bias criteria with 6 domains [7]: (1) random, (2) blinding of participants, doctor, and outcome assessors, (3) allocation concealment, (4) incomplete outcome data, (5) free of the suggestion of selective outcome reporting, and (6) informed consents. Discrepancies were resolved by consensus through discussion between the two reviewers.

## 3. Results

Six articles met our inclusion criteria (Table 1) [8–13]. The Cochrane reviews included were published between 2006 and 2011. The studies in these reviews mainly originated from China. They included between 3 and 18 primary studies. Four reviews were concerned with angina pectoris (unstable or stable) [9, 11–13], one review was concerned with heart failure [10] (heart failure was primary caused by CHD), and one review was concerned with acute myocardial infarction [8].

 Four Cochrane reviews concluded positively that TCHP may be or appears to be effective. Two reviews showed that the evidence is too weak to make conclusion. No reviews made definite conclusion. All reviews indicated that high-quality trials are required to assess the efficacy and safety of TCHP for CHD and the finding should be interpreted with care because of the very low methodological quality of studies and potential publication bias.

There are 69 studies in the six reviews. Two studies were reported from 1981 to 1985; one study was reported from 1986 to 1990; three studies were reported from 1991 to 1995; twenty-six studies were reported from 1996 to 2000; thirty-five studies were reported from 2001 to 2005; only two studies were reported from 2006 to 2011. Therefore, the most likely reason for the weak evidence of TCHP for CHD is the previous poor methodology.

The randomized clinical trials (RCTs) contained in four Cochrane reviews [8–10, 12] were mainly on the basis of conventional western medicine. But the basic treatment is not unchangeable. The RCTslisted in two Cochrane reviews [11, 13] directly contrasted one TCHP with western medicine or other TCHP. Two Cochrane reviews [8, 13] summarized different TCHP for CHD. The TCHP mentioned in these RCTs were injection (e.g., Shengmai Injection, Puerarin), oral Chinese patent medicine (e.g., Yi Xin Mai, Bao Xin Bao, Li Nao Xin, Shengmai Oral Liquid, Suxiao Jiuxin Wan, Tong Xin Luo), or Chinese herbal decoction. Four Cochrane reviews [9–12] summarized single TCHP for CHD.

In order to assess the status of the quality of the studies of TCHP, we also searched the CENTRAL in The Cochrane Library Issue 4 of 4 Oct 2011. Eight studies were included (Table 2) [14–21]. These studies primary originated from China. These studies were all making an explicit statement that the participants were randomly assigned to different groups, but two were not describing the details. Only four RCTS adopted the application of blinding: one did not report details [18] and three reported that the participants and doctors were blind [14, 19, 21]. One of the trials adopted allocation concealment [14]. Trials with inadequate blinding and inadequate allocation concealment may result in limited evidence. Six trials did well in the incomplete outcome data adequately addressed [14–16, 18, 19, 21]. Only one trial did well in the free of the suggestion of selective outcome reporting [18]. Not every trial made explicit statement that the participants signed the informed consents [16, 17, 19]. These RCTs had more participants than usual RCTs. They usually have 60 to 100 participants [15–18, 20, 21]; only 1 RCT has 35 participants [19] and 1 RCT has 859 participants [14]. These shortcomings highlight the importance of following CONSORT procedures in the future studies [22]. Anyway, the quality of primary studies was better than before, and we still need further progress.

## 4. Discussion

The current Cochrane reviews indicated the potential benefit of TCHP in treating CHD, but none of them drew a definite conclusion because of the poor quality of primary studies. Although Cochrane reviews have the reputation for being more transparent and rigorous than other systematic reviews, the conclusion needs further discussion. The RCTs listed in two reviews [8, 13] were not the same TCHP. The treatments in the control groups, and the durations of the RCTs were also varied. In addition, different TCHP applys to different syndrome according to TCM theory. All of these reviews did not involve this question.

Therefore, four reviews [9–12] about single TCHP are more persuasive. They all made the conclusion of “A,” indicating the TCHP may be or appears to be effective. The other two reviews made the conclusion of “B.” One review about “Danshen for acute myocardial infarction” concerned with the herb Danshen, but Danshen was not the only part of the treatment. Thus the heterogeneity of included RCTs cannot be ignored. The other review of “herbal products for stable angina” is concerned with three different TCHPs comparing with isosorbide dinitrate [13]. It also made the conclusion of “B,” indicating the evidence is insufficient and reliable conclusions could not be drawn.

In conclusion, although some Cochrane reviews have shown the potential benefit of TCHP in treating CHD, more evidence from high-quality trials is needed to support the clinical use of TCHP. However, well-designed randomized clinical trials of TCHP with rigorous methodology are in progress or have been completed at several institutions around the world [6]. We hope that the effectiveness and safety of TCHP can be confirmed in the near future.

## Figures and Tables

**Table 1:** Cochrane Reviews of TCHP for CHD.

First author	TCHP	Control group	Condition	Number of RCTs	Participants	Conclusion
Wu et al. [8]	Danshen as part of decoction	Different basic treatment	Acute myocardial infarction	6	2368	B
Wang et al. [9]	Puerarin	Different basic treatment	Unstable angina pectoris	20	1240	A
Zheng et al. [10]	Different forms of Shengmai	Different basic treatment	Heart failure	6	440	A
Duan et al. [11]	Suxiao jiuxin wan	Isosorbide dinitrate or nitroglycerin or other TCHP	Angina pectoris	15	1776	A
Wu et al. [12]	Tongxinluo	Different basic treatment	Unstable angina pectoris	18	1413	A
Zhuo et al. [13]	Different herbal products	Isosorbide dinitrate or other TCHP	Stable angina	3	216	B

Notes: RCT: randomized clinical trial.

A: TCHP may be or appears to be effective.

B: The evidence is insufficient, reliable conclusions could not be drawn.

**Table 2:** Registered Studies of TCHP for CHD in the Cochrane Central Register of Controlled Trials.

First author	TCHP	Condition	Participants	Random	Blinding of participants, personnel, or outcome assessors	Allocation concealment	Incomplete outcome data adequately addressed	Free of the suggestion of selective outcome reporting	Informed consents	Conclusion
Chu et al. [14]	Xuefu Zhuyu capsule	Unstable anginal patients after percutaneous coronary intervention	90	Yes but no details	Participants and doctor	Yes	Yes	Unclear	Yes	A+
Li et al. [15]	Specific TCHP	Myocardial perfusion in AMI patients after revascularization	80	Yes	Not mentioned	Not mentioned	Yes	Unclear	Yes	A+
Li et al. [16]	Specific TCHP	Ventricular wall motion in AMI patients after revascularization	80	Yes	Not mentioned	Not mentioned	Yes	Unclear	Unclear	A
Hu et al. [17]	Shenfu injection	Heart function in patients with chronic heart failure	63	Yes but no details	Not mentioned	Not mentioned	Unclear	Unclear	No	A+
Tam et al. [18]	Salvia miltiorrhiza and Pueraria lobata	Vascular function and structure in coronary patients	100	Yes	Double-blind but no details	Not mentioned	Yes	Yes	Yes	A+
Qiu et al. [19]	Specific TCHP	The clinical symptoms and quality of life of the AMI patients undergoing PCI	35	Yes	Participants and doctor	Not mentioned	Yes	No	Unclear	A+
Fan et al. [20]	Qihong decoction	Rehabilitation of patients after coronary artery bypass	72	Yes	Not mentioned	Not mentioned	No	No	No	A+
Wang et al. [21]	Shenshao tablet	The quality of life for CHD patients with UA	66	Yes	Participants and doctor	Not mentioned	Yes	Unclear	Yes	A+

Notes: A+: TCHP has definitely effect.

A: TCHP may be or appears to be effective.
